# Social determinants associated with mental health problems in youth with intellectual disability: a systematic literature review

**DOI:** 10.1007/s00787-025-02794-7

**Published:** 2025-07-01

**Authors:** M. M. C. Storm, W. M. van Eldik, L. A. Nooteboom, R. R. J. M. Vermeiren

**Affiliations:** 1 LUMC Curium–Department of Child and Adolescent Psychiatry, Leiden University Medical Center, Post Box 15, 2300 AA Leiden, The Netherlands; 2 Parnassia Group, Youz, De Banjaard, The Hague, The Netherlands; 3 Parnassia Group, Youz, The Hague, The Netherlands; 4 Department of Psychiatry, Leiden University Medical Center, Leiden, The Netherlands

**Keywords:** Intellectual disability, Mental health, Social determinants, Children

## Abstract

Individuals with intellectual disability (ID) face a higher risk for developing mental health problems. Recent research emphasizes the relevance of social determinants of mental health (SDOMH) in relation to this risk. This review aims to synthesize evidence on the associations between SDOMH and mental health in youth with ID, focusing on risk and protective factors across demographic, economic, social, and neighborhood domains. A systematic search of multiple databases identified 51 relevant studies, including 36 cross-sectional and 15 longitudinal studies. Using a framework-led approach, the strongest evidence emerged from the social/cultural domain (*n* = 46), followed by economic (*n* = 17), demographic (*n* = 8), and neighborhood (*n* = 1). A key finding is the substantial variability and mixed results across studies, highlighting complex, context-dependent associations. Nonetheless, some patterns emerged within the social/cultural domain. Based on both cross-sectional and longitudinal evidence, the most consistent links were found between social and cultural stressors and mental health vulnerabilities. Specifically, in families where children with ID displayed more behavioral problems, 1) parents experienced high distress or internalizing problems; 2) parenting behaviors were more negative; and 3) children faced stressful life events. The review also reveals notable research gaps, including limited attention to broader environmental and neighborhood factors, few longitudinal and multivariate studies, and minimal focus on protective factors. Future research should adopt a systematic approach, prioritize underexplored environmental and protective factors, apply longitudinal and multivariate designs to examine causal pathways, incorporate qualitative methods, and standardize measures to enable consistent analysis across studies.

**Supplementary Information:**

The online version contains supplementary material available at 10.1007/s00787-025-02794-7.

## Introduction

Individuals with intellectual disability (ID) are at elevated risk of experiencing mental health problems [1]. As traditional research has centered on exploring biological or individual demographic factors contributing to these problems [2], recent studies emphasize the significant role of a comprehensive set of socio-demographic factors across different life domains [3, 4]. This shift acknowledges the associations between mental well-being and aspects of the social, economic, and cultural environments, collectively referred to as social determinants of mental health (SDOMH) [5, 6]. SDOMH represent the structural conditions individuals encounter throughout life, including where they live (e.g. housing), work (e.g. employment), and age (e.g. neighborhood conditions) [4, 7, 8]. Compelling evidence is increasingly connecting these SDOMH to the likelihood of experiencing mental health problems [9].

Previous studies demonstrate that encountering adverse SDOMH early in life can have significant implications for later mental well-being [7, 10, 11]. This might especially be relevant for youth with ID, since they are at a greater risk of facing unfavorable SDOMH during their lifetime [12]. In fact, these children bear a dual burden. First, youth with ID are particularly vulnerable to environmental disadvantages, placing them at a heightened risk of occupying a lower social stratum [13]. For instance, studies revealed that youth with ID are more often raised in disadvantaged households compared to their typically developing peers [13, 14]. Such social positioning is associated with elevated levels of cumulative stress, which may contribute to greater mental health vulnerabilities [7]. Second, there are inherent limitations associated with ID itself, complicating their ability to adapt and cope with difficulties. For instance, children with ID may have difficulty communicating distress and regulating their reactions to their environments, which has been linked to internalizing or externalizing mental health symptoms. In physically adverse environments, such as overcrowded noisy areas, these challenges can become even more pronounced, making them more vulnerable to stress [5]. Thus, the ID itself adds another layer of complexity to the ability to manage stress and adversity, which may be linked to a higher likelihood of mental health problems for these children.

Given the potentially significant role of adverse SDOMH for the mental well-being of youth without ID [7, 15], it is surprising that empirical evidence regarding children with ID is sparse. To date, two reviews have synthesized findings on factors related to mental health problems in children with ID [16, 17]. One mainly focused on individual demographic variables, such as age, gender, and level of functioning [16]. The other examined a limited range of contextual SDOMH, including parental psychopathology, stress, family functioning, single-parent households, and socio-economic status (SES) [17], which were more consistently linked to child psychopathology. Together, the reviews provided a first foundation for understanding the role of some SDOMH. However, these reviews were restricted in their scope, as they did not investigate associations between psychopathology and structural conditions children encounter throughout life such as neighborhood conditions and social support. A more recent review on risk factors for developing ID, rather than mental health problems, did focus on additional SDOMH such as various environmental factors, including geographical remoteness, air pollutants, and soil concentration [18]. This shift reflects a growing recognition of the broader environmental SDOMH. Therefore, in this review, we have chosen to expand the scope to include a focus on broader social and environmental factors related to mental health in children with ID. Addressing these factors is important for developing a more comprehensive understanding of the interconnectedness between contextual characteristics and mental health in children with ID.

Accordingly, this literature review aims to synthesize existing empirical knowledge about associations between SDOMH and mental health problems in youth with ID, focusing on both risk and protective contextual factors. To provide a comprehensive overview and enhance the understanding of the role of different SDOMH, we will summarize, analyze, and categorize them based on the theoretical framework of Lund et al. (2018), which outlines several overarching domains of SDOMH, including the demographic, economic, social/cultural, and neighborhood domain. Drawing on the concept of a dual burden, we generally expect that adverse SDOMH across all domains will be linked to greater mental health problems in youth with ID. Specifically, since prior literature reviews suggest that poorer parental mental health, higher family stress, and a dysfunctional home environment are associated with increased mental health problems in children with ID [17], we anticipate particularly strong associations in the social/cultural domain. For other SDOMH domains (demographic, economic, and neighborhood), where reviews have provided inconsistent or limited evidence, we take a more exploratory approach to identifying potential associations. By synthesizing this knowledge, our goal is to support a more nuanced understanding of the associations between SDOMH and mental health in youth with ID—informing the development of comprehensive, evidence-based strategies for identifying patterns of vulnerability and addressing the needs of affected families.

## Method

**Fig. 1:**
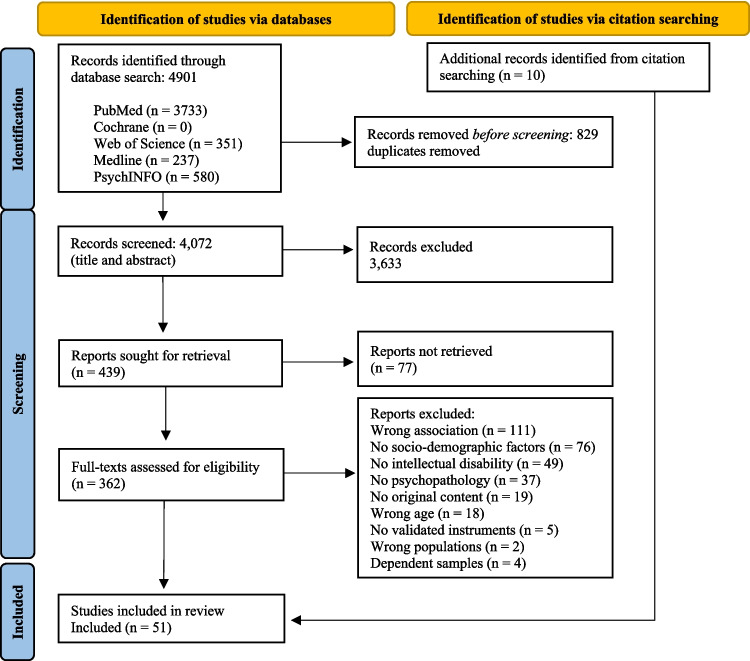
PRISMA flow diagram

A record of the current research protocol was prospectively registered in the International Database of Prospectively Registered Systematic Reviews in Health and Social Care (PROSPERO, registration number CRD42022334214) following the Preferred Reporting Items for Systematic Reviews and Meta-Analysis (PRISMA) guidelines [19]. The study selection process was a stepwise procedure based on the PRISMA flow diagram (Fig. 1).

### Search strategy

The present review received assistance from a medical research librarian from Leiden University Medical Centre in formulating the search strategy. Subsequently, a literature specialist from Parnassia Groep Academia with extensive knowledge about the subject performed a final check on the search strategy. A systematic search was conducted by consulting the following electronic databases: PubMed, PsycINFO, MEDLINE, Cochrane Library, and Web of Science.

The search was focused on the following four predefined categories: (A) ID (including mental retardation, learning disability, and intellectual deficit), (B) youth (including infant, child, adolescent, and young adult), (C) psychopathology (including both internalizing and externalizing problems, as well as developmental disorders such as Attention Deficit Hyperactivity Disorder (ADHD) and Autism Spectrum Disorder (ASD)), and (D) SDOMH, defined as circumstances that affect an individual's health condition, as outlined by the World Health Organization [4]. The conceptual theoretical framework presented by Lund et al. (2018) guided our selection of relevant terms for SDOMH [6], supplemented by input from a librarian or literature specialist, and prior literature [3]. We tailored this framework, originally consisting of five domains: demographic, economic, social/cultural, neighborhood and environmental events [6], to align with our study's objectives. This modification ensured that the framework addressed the unique circumstances of youth with ID, with a specific emphasis on the developmental perspective prevalent among children in Western countries. Consequently, we incorporated parental and family factors across various subdomains to better integrate this proximal environment associated with childhood mental health. For instance, parental ethnicity (i.e., demographic domain) and parental education (i.e., social/cultural domain) were included as they reflect the structural and contextual characteristics that shape a child's environment. When needed, we held extensive discussions among co-authors to ensure a clear and systematic classification of all factors. Moreover, we omitted the domain of environmental events, including war and natural disasters, as they are extreme circumstances with limited occurrence in industrialized societies. Appendix A illustrates the theoretical framework, providing examples of factors within each domain for clarity.

The complete search strategy with specific search queries for each database can be found in Appendix B. All identified studies were imported to the bibliographic reference manager Endnote® (X9). Additionally, the reference lists of the included studies were searched manually to identify potentially relevant articles that were missed during the computerised search. The final search was executed on September 5th, 2024.

### Eligibility Criteria

To be included in this study, records had to meet the eligibility criteria presented in Table 1.

**Table 1:** Summary of inclusion and exclusion criteria

Study characteristic	Inclusion criteria	Exclusion criteria
Participants	Focus on study samples including youth aged 0 to 23 years old to capture childhood through late adolescence, with a mean age under 23 years Diagnosed with ID, encompassing all severity levels (mild, moderate, severe, profound) in accordance with DSM-5 criteria [20]	Samples with < 80% diagnosed with ID
Outcome	Must report on mental health problems. Mental health problems were assessed via validated instruments or meeting diagnostic criteria [21]	-
Risk and protective factors	Report on SDOMH, fitting the framework of this study	Individual characteristics, such as child’s age, gender and ethnicity
Association	Investigating the relationship between SDOMH and mental health problems in youth with ID	-
Study design	All study designs were accepted, including qualitative, quantitative, and mixed-method approaches	-
Publication type	Peer-reviewed manuscripts in English or Dutch, available as full-text articles	Publications such as conference abstracts or position papers
Publication year	No restrictions on the year of publication	-

### Study selection

The study selection process followed a stepwise procedure. Initially, our database search yielded 4,901 studies, with 3,583 non-duplicate publications. Subsequently, the remaining studies were transferred to Rayyan software for labeling and selection [22]. Two independent reviewers (MS and WE) evaluated the studies based on their titles and abstracts, guided by predefined inclusion and exclusion criteria. Any disagreements between the researchers were resolved through discussion to achieve consensus. The inter-rater agreement for this screening round, measured by Cohen’s Kappa, was 0.85, indicating almost perfect agreement between the two reviewers [23]. Then, the first reviewer (MS) assessed the full text of the remaining articles, with any uncertainties resolved in a meeting with the second independent reviewer (WE). Figure 1 illustrates an overview of the study selection procedure.

### Data extraction and synthesis

As a foundation for data extraction, we extended the Cochrane Data Extraction Template with additional relevant factors and pilot tested the form on ten randomly selected publications. Then, MS reviewed the eligible research articles and extracted key information into a data extraction table, including the title, authors, publication year and type, study description, methodology, and sample demographics. Any doubts regarding the eligibility of research articles were addressed and resolved during a meeting with WE to reach a consensus. To prevent publication bias, all studies were screened for using identical datasets. Among these, four studies shared identical datasets. In such cases, the longitudinal study was preferred over the cross-sectional. If both studies had the same design, the study including most SDOMH was chosen. After completing the data extraction, we categorized the SDOMH into four main domains: demographic, economic, social/cultural, and neighborhood. A synthesis of the findings was then conducted to evaluate the evidence within each domain. A detailed overview of the study characteristics can be found in Appendix C.

### Quality appraisal

Individual study quality was assessed using the Appraisal Tool for Cross-Sectional Studies (AXIS) [24], specifically designed for non-experimental research. AXIS includes 20 items assessing key elements such as sample size justification, use of validated measures, and statistical methods. Prior to assessment, each of the 20 questions was assigned a weighting score (1, 1.5, or 2) based on the researchers mutually agreed-upon perception of its importance. Scores were assigned for questions answered affirmatively ('yes'), except for two items that were reverse-coded based on their wording. Subsequently, after the assessments, these scores were aggregated to generate a total score per article, ranging from 0 to a maximum of 26. As AXIS lacks fixed cut-off scores, we established thresholds for the total scores based on the distribution of quality appraisal scores across the included studies (range: 6–26), to create a relative approach to assessing study quality. A complete list of the AXIS items and their assigned weights is provided in Appendix D. The scores were divided into three equal tertiles and based on this distribution, studies were categorized as high quality (≥ 19 points), medium quality (14–18 points), and low quality (≤ 13 points). Two authors independently evaluated each study, resolving discrepancies through consensus or involving a third author, if necessary, to ensure a robust evaluation process. A detailed overview of the critical appraisal scores per domain can be found in Appendix E.

### Strength of evidence

To clarify which domains were characterized by substantial evidence and which by insufficient evidence [25, 26], we evaluated the strength of evidence per domain. This method facilitates a comprehensive comparison of multiple studies across demographic, economic, social/cultural, and neighborhood domains, allowing for a relative assessment of overall quality using five predefined criteria.

- *Size of Evidence:* The strength of evidence was determined by the number of studies within each domain. Domains with 38 or more studies (≥ 75% of the total reviewed) were classified as substantial (+); those with 13 to 37 studies (25–75%) as moderate (±); and those with fewer than 12 studies (< 25%) as limited (−).
- *Quality of studies:* Based on the quality assessment for individual studies, the overall quality of the domain was assessed. A high rating (+) was assigned to domains where over 75% of studies were high quality; medium (±) for 25 to 75%; and low (−) where less than 25% were rated as high quality.
- *Consistency of findings:* Consistency of findings was assessed across domains, with results classified as consistent (+), mixed (±), inconsistent (−), or contradictory (− −). Findings were considered consistent when all studies within the same domain supported similar conclusions. Results were classified as mixed when at least one study, compared to the others, focused on different subpopulations and produced varying results. Findings were labelled inconsistent when two studies on the same subpopulation produced different results, with one finding an association and the other not. Finally, findings were deemed contradictory when at least two studies on the same subpopulation produced opposing results, such as one study finding a positive association and another finding a negative association.
- *Context:* Each domain's context was classified as either mixed or specific. Mixed contexts (+) reviewed studies where results were observed across a diverse population, such as general community samples spanning a variety of psychological disorders. Specific contexts (−) were designated for domains focusing on a particular sample, such as specific groups of children with a syndrome or the focus on a specific type of mental health problem.
- *Perspective (source of evidence):* SDOMH or child’s mental health problems based on evidence from two or more perspectives (informants), including youth, parents, and teachers, were deemed multiple (+), while SDOMH or child’s mental health problems relying on evidence from a single perspective were labeled single (−).

The overall strength of evidence was based on cumulative scores across five criteria: size of evidence, study quality, consistency of findings, context, and perspective. For each criterion, the evidence was rated using the following scale: + (positive), ± (mixed), − (negative), or − − (contradictory; only applicable to consistency of findings). For the cumulative scoring, these ratings were translated as follows: + = 1 point, ± = 0.5 points, − = 0 points, and − − = 0 points. The strength of evidence then was classified into the following categories: very strong (5 points), strong (3.5–4.5), medium (2–3), limited (0.5–1.5), or no evidence (0).

## Results

### Study characteristics

This review included 51 studies − 36 cross-sectional and 15 longitudinal − mostly conducted in the USA and UK. The studies covered samples across a wide age range, mainly focusing on early to middle childhood, with some also including adolescence and multiple age stages. Sample sizes varied significantly, from 17 to 10,438 participants (*M* = 892, *SD* = 2,055). Most studies showed a male gender imbalance, with male participation ranging from 44% to 88.2%. The majority of studies addressed a wide range of ID severities, covering at least three levels (e.g., mild, moderate, and severe). However, 14 studies did not specify the severity level of ID. Four studies (8.2%) focused on specific syndromes, such as Down and Fragile X syndrome (FXS). Regarding mental health problems, the most common were behavior problems (*n* = 16), followed by ASD symptoms (*n* = 14), unspecified psychopathology (*n* = 9), internalizing and/or externalizing problems (*n* = 7). Less common were anxiety (*n* = 3), depression (*n* = 3), conduct disorder (*n* = 3), hyperactivity (*n* = 2), ADHD symptoms (*n* = 2), emotional problems (*n* = 3), and maladaptive behavior (*n* = 2). Notably, 37 out of 51 studies (72.5%) used only univariate analyses, and no qualitative studies were identified in the search. Critical appraisal classified 19 studies as high quality, 14 as medium, and 18 as low. High-quality studies were primarily studies with clearly defined research objectives, justified sample sizes, validated measures for mental health problems, and transparent reporting of statistical methods. A detailed overview of study characteristics is provided in Appendix C.

### Outcomes

The aim of this review was to synthesize evidence on the associations between SDOMH and mental health problems in youth with ID using a framework-led approach [27]. A great variety of SDOMH was found within the studies, which led us to categorize them into subdomains within our four main domains. Moreover, we explored whether associations within each SDOMH domain varied across subgroups based on ID severity, child age, and type of mental health problems. Table 2 summarizes the strength of evidence for each SDOMH domain, excluding the neighborhood domain, which had only one study and could not be assessed.

**Table 2:** Summary of strength of evidence per domain

Domain	Size of evidence (no. of studies)	Quality (individual studies)	Consistency of findings	Context	Perspective	Overall strength of evidence
Demographic	-	±	-	+	+	Medium
Economic	±	±	--	+	+	Medium
Social/cultural	+	±	--	+	+	Strong
Neighborhood	N.A	N.A	N.A	N.A	N.A	N.A

+ = substantial evidence/high quality/consistent findings/mixed contexts/multiple perspectives; ± = moderate evidence/medium quality/mixed consistency; - = limited evidence/low quality/inconsistent findings/specific contexts/single perspective; -- = contradictory findings; N.A. = not applicable

### Associations across subgroups of ID severity, age and type of mental health problem

To examine potential patterns within subgroups, studies were categorized based on ID severity, including individual levels (severe, moderate, mild, and borderline) as well as combined severity categories. Table 3 in Appendix F provides an overview of studies across these ID severity levels within each domain. This table presents the number of studies for each level or combination of ID severity across domains, indicating how many reported at least one significant result. Studies were fairly distributed across severity levels, with the social/cultural domain most consistently examined and yielding the highest proportion of significant findings. However, no consistent pattern of significant associations between SDOMH and mental health problems emerged across domains, indicating that specific domains were neither more frequently studied nor more strongly associated with mental health issues in any ID severity group.

Studies were then grouped based on age categories, including individual stages (early childhood [0–6 years], middle childhood [6–12 years], early adolescence [12–16 years], and late adolescence [16 + years]) as well as combined age groups. As shown in Table 4 of Appendix F, similar to findings by ID severity, there was no clear indication that specific domains are either more frequently studied or more closely linked to mental health issues across different age groups.

Finally, due to substantial variability in types of mental health problems reported and the absence of specified mental health conditions in some studies, conducting subgroup analyses for all mental health conditions was not feasible. We therefore focused on the two most frequently reported outcome groups—ASD and externalizing problems—based on a subset of 41 articles. Externalizing problems included studies reporting on outward-directed behaviors such as aggression, hyperactivity, conduct issues, and general behavioral problems. These conceptually similar constructs were grouped together to allow for meaningful comparison. As shown in Table 5 of Appendix F, the social/cultural domain was again the most frequently studied and showed the highest proportion of significant associations for both ASD and externalizing problems. However, the proportion of significant findings was greater for externalizing problems than for ASD within this domain. A similar trend was observed in the economic domain, despite the smaller number of studies overall. In contrast, ASD showed more significant associations in the demographic domain, relative to externalizing problems.

### Domain 1. Demographic

The demographic domain included SDOMH reflecting general population characteristics, which were divided into two distinct subdomains: parental ethnicity and parental age. A total of eight studies examined these factors.

#### Parental ethnicity

Five studies examined associations between parental ethnicity and mental health problems in children with ID, including four cross-sectional and one longitudinal. The findings were mixed. Among the four cross-sectional studies, one reported significant differences in maternal race/ethnicity between children with ID only (higher proportion of non-Hispanic black mothers) and those with both ID and ASD [28]. However, three other studies reported no association between ethnicity and problem behaviour in children with ID [29–31]. Additionally, one longitudinal study focused on maternal migration rather than ethnicity directly, finding that children born earlier than four years before maternal migration were less likely to have ID with autism compared to those born in the year following maternal migration [32].

#### Parental age

Four cross-sectional studies examined the relationship between parental age and mental health problems in children with ID, though findings were inconsistent. One study found that mothers of children with both ID and ASD were significantly younger than those of children with ID only [28]. Another reported no age differences [33], while a third found that maternal age was not associated with emotional or conduct problems, but that older maternal age was linked to more hyperactivity [34]. A fourth study found no association between parental age under 18 and behavior problems in young children with developmental delays [35].

### Domain 2. Economic

Within the economic domain, seventeen studies examined the role of economic factors in relation to mental health problems in children with ID. To synthesize the results, the economic factors were categorized into three subdomains: family income, income-related factors, and composite SES measures.

#### Family income

Four cross-sectional and two longitudinal studies examined a direct relationship between family income and mental health problems in children with ID, yielding inconsistent results. Whereas three cross-sectional studies reported no link between family income and mental health issues [36–38], one cross-sectional study found that children with ID from lower-income households were more likely to have psychiatric disorders [39]. Longitudinally, one study found no link [40], whereas the other study reported slower income growth over eight years in families of children with both ID and ASD compared to families with children who have only ID [41].

#### Income related factors

Six studies–five cross-sectional and one longitudinal–examined income-related factors and mental health in children with ID, with mixed results. Cross-sectional evidence indicated that household poverty was significantly associated with increased behavioral problems in children with ID [42]. Consistently, another study found that mothers of children with severe ID and behavior problems felt a greater need for financial help [43]. However, three studies found no significant associations between income-related factors, such as rented accommodation or reliance on benefits [44], Family Affluence Scale scores [45], and health insurance coverage [46], and mental health problems in children with ID. The longitudinal study found that over eight years, families with children with ID and ASD paid significantly lower federal taxes than those with only ID [41].

#### Composite SES measures

Six cross-sectional studies investigated composite SES measures, including financial hardship and socio-economic position. Remarkably, half of these studies did not specify how SES was measured. The results were either inconsistent or contradictory. In one UK study low socio-economic position, measured by household income, occupational prestige, and maternal education, was associated with more behavior problems in children with developmental delays [35]. Similarly, in another sample, lower SES (i.e., unspecified measures) was associated with increased behavior problems in children with ID [47]. In contrast, another study found that families of children with only ID had significantly lower SES compared to those with both ID and ASD, based on education level, occupation, employment status, and total household income [48]. Contrastingly, three studies found no significant link between SES and mental health problems in children with ID [49–51].

### Domain 3. Social/Cultural

The social/cultural domain covers the broadest range of factors related to the mental health of children with ID. These factors are grouped into five subdomains: parental well-being, employment and education level, parent–child relationship, family dynamics, and life events. A total of 46 studies explored these associations.

#### Parental well-being

Parental well-being was reported in 23 studies, encompassing six different subcategories of well-being: general mental health, (di)stress, internalizing problems, substance use, maternal somatization and maternal life satisfaction. Each subcategory yielded results that were either mixed or inconsistent.

Ten studies, including eight cross-sectional and two longitudinal, examined the association between parental mental health and the mental health of children with ID. Cross-sectionally, six studies reported a link between lower parental mental health and increased psychological problems in children [31, 35, 39, 51–53]. Caregiver mental health problems were associated with higher rates of psychiatric diagnoses in children [39, 53], particularly in boys [29], and to increased depression and severe behavioral issues [51, 52]. Contrastingly, two studies found no significant difference in parental mental health between parents of children with and without behavioral problems [54, 55]. Longitudinally, both studies found that parental mental health problems were linked to increased psychopathology in children with ID one year later. One study associated parental mental health treatment history with variations in internalizing and externalizing problems [56], whereas the other found that parental mental health issues predicted psychiatric disorders in children [29].

Ten studies–five cross-sectional and five longitudinal–consistently linked greater parental (di)stress to increased behavior problems in children with ID, with seven focusing on maternal distress [42, 57–62]. Cross-sectionally, all five studies found at least one significant association between greater parental distress and increased child behavior problems [42, 58, 59, 61, 62], though links with having ASD were not significant [62]. Longitudinally, all five studies found a bidirectional relationship between parental distress and child behavior problems over time [57, 60], particularly for children's externalizing problems [56, 63, 64]. However, some associations, such as overall psychopathology [56], were not significant.

Eight studies investigated parental internalizing problems, encompassing depression (n = 7), anxiety (n = 3), and general internalizing problems (n = 1). Among the seven studies on parental depression, five were cross-sectional and two were longitudinal. Cross-sectionally, three studies found that higher maternal depression was associated with increased mental health problems in children with ID, specifically ASD [36], child depression [52], and maladaptive behavior [59], whereas two studies reported no significant link with behavioral problems [31, 54]. Longitudinally, one study found that higher maternal depression levels predicted increased internalizing and externalizing problems in children over time [65], whereas another study reported no association with internalizing problems [64]. Of the three studies on parental anxiety, two were cross-sectional, both showing that higher anxiety in parents was linked to more behavioral or psychiatric issues in children with ID [31, 36], whereas the longitudinal study found no significant link with internalizing or externalizing problems [64]. Regarding general parental internalizing problems, one cross-sectional study found no association with FX syndrome and autism but linked higher maternal internalizing symptoms to behavioral problems in adolescents and adults with FX syndrome [37].

Parental substance use was examined in two cross-sectional studies. One found that parental addiction was linked to increased externalizing symptoms in children with ID [66], whereas the other found no link between parental alcohol or drug abuse and behavior problems in children with developmental delay [35].

Two studies linked higher maternal somatization to increased behavioral problems in children with ID. A cross-sectional study found this among Latina caregivers of children with maladaptive behavior [59], whereas a longitudinal study identified maternal somatization as a significant predictor of increased behavioral problems over two years, particularly in mothers of children with both ID and ASD [57].

Maternal life satisfaction was examined in two studies. One cross-sectional study found no link with behavioral problems in children with ID [42]. A longitudinal study similarly reported no association over an eight-year period [63].

#### Employment and education level

Employment and education levels were reported in 19 studies, divided into four subcategories: child employment, parental employment, parental education, and a combination of parental employment and education. The findings within each subcategory were mixed, inconsistent or contradictory.

One longitudinal study explored employment among young adults with ID and found that those in open employment for two years had a decline in behavior problems, whereas those in training, sheltered employment or day recreation programs, showed no change [67].

Nine studies examined parental employment and child mental health, comprising seven cross-sectional and two longitudinal. Among the cross-sectional studies, two found that unemployment or lower job status correlated with more child behavior issues [39, 49] and ASD [49]. Consistently, children with ID from lower social classes were more likely to have conduct disorders, ADHD, and autism [39]. Inconsistently, three studies found no link between maternal employment and child behavior problems [30, 33, 36], nor did two others find a link between occupational prestige or social class and behavior disorders [38, 55]. Longitudinally, one study found that low SES did not predict psychopathology but was linked to increased internalizing problems [56], whereas another reported no association between parental social class, employment status, and child psychopathology over time [68].

Nine studies examined the link between parental education and child behavior problems, covering seven cross-sectional and two longitudinal. Cross-sectionally, two studies found that lower parental education was associated with increased behavior problems [34, 38]. Specifically, children of mothers without qualifications showed more conduct and emotional disorders [38]. Contradictory, one study found a reverse relationship, where higher maternal education was more common among mothers of children with both ID and ASD [28]. Four cross-sectional studies reported no association between parental education levels and behavior problems in children with ID [33, 36, 37, 44]. Longitudinally, one study found that lower parental education predicted increased disruptive disorders over one year but not DSM-IV diagnoses, anxiety, or mood disorders [29], whereas another found no evidence that parental education predicted child psychopathology over time [40].

Three studies, comprising two cross-sectional and one longitudinal, examined a composite measure of parental education and occupational level. Cross-sectionally, two studies found that lower parental education, unemployment, and unskilled work were associated with higher rates of psychopathology [69] and greater internalizing and externalizing problems in children with ID [70]. Longitudinally, one study found that lower SES was linked to increased internalizing problems over one year but did not predict total psychopathology [56].

#### Parent–child relationship

The parent–child relationship was examined in 14 studies, split into positive and negative subcategories, yielding inconsistent results.

Seven studies–four cross-sectional and three longitudinal–examined positive elements of the parent–child relationship, including parental behaviors, feelings, and dyadic interactions. Findings varied depending on the type of mental health problem studied. Cross-sectionally, three studies found inverse links between these positive relationship elements and mental health problems [47, 52, 54]. A higher parental sense of competence was associated with less behavioral problems [47, 54] and lower depression levels [52], whereas higher attachment levels were linked to lower depression but not behavioral problems [52]. Remarkably, positive discipline (i.e., structured, corrective consequences rather than punitive or harsh measures) was associated with higher externalizing behaviors [47]. Longitudinally, some positive parent–child interactions were linked to fewer behavior problems over time, though effects varied by age and behavior type. Early positive parenting was associated with reduced behavioral issues in childhood, but some effects did not persist [71]. Maternal warmth and scaffolding were generally linked to better behavioral outcomes, though their impact differed across behaviors and conditions [40, 65].

A total of 14 studies, encompassing 10 cross-sectional and four longitudinal–examined negative parenting behaviors, such as hostility, overprotection, and negative parental feelings. Cross-sectionally, seven studies linked these behaviors to mental health difficulties [35, 36, 39, 44, 47, 66, 72], with harsh discipline consistently associated with behavioral problems [39, 44, 47] and with ASD diagnoses [36]. Emotional abuse, parenting difficulties, and parental criticism were associated with increased behavioral issues [35, 44, 64, 66], but one study found that overall maltreatment rates—including emotional abuse—did not differ between children with ID and those with both ID and ASD [73]. Maternal overprotection was linked to child anxiety, but paternal overprotection was not [72]. Varying results were found for negative parental feelings, with role restriction unrelated to behavioral problems [54] or depression [52], whereas discontinuity in care was linked to behavior issues [44]. Longitudinally, maternal criticism and adverse parent–child relationships were linked to lasting behavioral problems, though effects varied. Criticism predicted more severe externalizing symptoms and behavior problems [65], whereas another study found no link [64]. Further, no long-term effects on internalizing or autism symptoms were observed. Mothers of children later diagnosed with ADHD exhibited more negative parenting and dyadic conflict [40]. Early adversarial parenting contributed to later conduct problems but did not persistently impact emotional difficulties, hyperactivity, or overall behavior [71].

#### Family dynamics

Various family dynamic factors were reported in 29 studies, which can be divided into the following four subcategories: family structure, the interparental relationship, family functioning, and social networks. Each subcategory yielded results that were either mixed, inconsistent or contradictory, illustrating the scattered nature of findings.

Family structure, examined in 16 studies, included marital status (*n* = 13), family size (*n* = 3), and birth order (*n* = 4). Regarding marital status, ten studies were cross-sectional and three were longitudinal. Cross-sectionally, six studies found an effect, though the findings were contradictory. Three studies linked single parenthood to higher risks of psychiatric and behavioral problems [38, 39, 43], whereas the other three showed that mothers of children with ID and dual diagnoses were more likely to be married [28, 48, 49]. The other studies found no significant associations between marital status and behavioral outcomes [30, 34, 36, 44]. Longitudinally, single parenthood predicted externalizing and internalizing problems but was not linked to overall psychopathology [56]. Mental health trajectories over time did not significantly differ between adolescents from single- and two-parent households [74], nor was nonfamily care associated with increased psychopathology risk [68, 74]. Regarding family size, no associations with behavioral problems were found among the three cross-sectional studies [35, 43, 69]. Results on the role of birth order on mental health in children with ID varied, with three cross-sectional studies and one longitudinal. Cross-sectionally, being the youngest sibling was linked to increased hyperactivity [34] but not to emotional, conduct problems, or overall psychopathology [69]. Longitudinally, being the youngest sibling was associated with increased behavioral problems over time [75].

Of six cross-sectional studies on the interparental relationship, three found that higher marital satisfaction was linked to lower levels of behavior problems or depressive symptoms in children with ID [52, 54, 61]. In contrast, three studies found no such associations: one reported no link between parental marital satisfaction and children with FX syndrome and autism [37], another found no association between marital quality and severe behavior disorders in adulthood [55], and no differences in parental conflict were linked to children's behavioral problems [76].

Family functioning was examined in 11 studies across five aspects: family dysfunction (*n* = 3), family cohesion (*n* = 2), domestic violence (*n* = 2), family quality of life (*n* = 2), and sibling factors (*n* = 3). Regarding family dysfunction, one cross-sectional study found that children with ID from families with more unhealthy functioning were more likely to have a diagnosed, emotional, or anxiety disorder [39]. The two longitudinal studies showed that family dysfunction predicted disruptive disorders but not anxiety or mood disorders [29] and uniquely contributed to total psychopathology, externalizing, and internalizing problems [56]. Two cross-sectional studies showed that family cohesion was not associated with behavioral problems in children with ID [37, 76]. Regarding domestic violence, defined as exposure to physical or verbal violence within the family setting, one cross-sectional study associated witnessing domestic violence with more externalizing symptoms in children with ID [66], whereas another cross-sectional study found no such link for children with ID [35]. Family quality of life was not linked to challenging behaviors or psychiatric diagnoses, but it was lower for youth with ASD or maladaptive behavior based on cross-sectional evidence [51, 62]. Regarding sibling factors, cross-sectionally, sibling mental health difficulties were linked to co-occurring psychiatric diagnoses [53]. Longitudinally, sibling referral to mental health care did not predict disorders after one year [29], and neither sibling warmth nor conflict predicted behavior problems or disorders [75].

Five cross-sectional studies examined social networks, focusing on social support (*n* = 3) and parental isolation (*n* = 2). Regarding social support, one study found that children with both ID and ASD had significantly worse family and friend relationships compared to those with ID alone [77]. Another found no link between social support and either ASD or challenging behaviors [62]. A third study showed that higher perceived social support from family, friends, teachers, and the community was associated with fewer emotional and conduct problems, but not hyperactivity [34]. As for parental isolation, one study found that parents of children with ID and behavioral problems were more socially isolated than those without such problems [54], though this isolation was not linked to the presence or severity of the child's depression [52].

#### Life events

Eight studies–six cross-sectional and two longitudinal–examined the association between life events and mental health, mostly focusing on negative or stressful experiences, with mixed results. Cross-sectionally, children with ID experiencing more stressful events were generally more likely to be diagnosed with psychiatric disorders [39, 51, 54, 66, 78], though one study found no significant association [35]. Longitudinally, one study found that negative life events predicted DSM-IV disorders, including mood disorders, after one year [29], whereas another showed that life event exposure uniquely contributed to internalizing problems but not to total psychopathology or externalizing problems [56].

### Domain 4. Neighborhood

Theoretically, the neighborhood domain includes environmental factors related to the area in which a family lives, such as neighborhood deprivation and violence, access to recreational facilities, and availability of services. One cross-sectional study addressed this domain and found that living in a violent neighborhood was linked to increased externalizing problems in children with ID [66].

## Discussion

This is the first review to comprehensively synthesize findings on the association between a wide range of social determinants of mental health and mental health problems in children with ID, expanding on previous reviews with a narrower focus [16, 17]. In doing so, this review highlights the diverse environmental contexts that are associated with variations in mental health problems among youth with ID. Using an existing framework to categorize SDOMH [6], we identified significant variability in both the types of SDOMH studied in relation to mental health problems and the findings across these studies. Consequently, the substantial heterogeneity and mixed results across studies indicated that drawing firm conclusions may be premature. Associations appear complex, context-dependent, and varying by individual child characteristics and across different life domains. Nonetheless, findings within the social/cultural domain generally aligned with our expectations. As anticipated, more adverse SDOMH in this domain were generally related to greater mental health problems in children with ID, supporting the idea that social and cultural stressors are associated with increased mental health vulnerabilities. Our exploratory approach to other life domains revealed less consistent patterns and important gaps, highlighting the need for further investigation into the interplay between multiple environmental stressors and mental health in this population.

This review encompasses studies on youth with a broad spectrum of ID severities, including specific groups such as children with Down syndrome and FXS, while also capturing the diverse expressions of mental health problems. Even more, the studies spanned different age groups, from early childhood to young adulthood. By not focusing on a single mental health problem, this review provided a broader understanding of how different mental health problems in youth with ID are connected to SDOMH, aiming to uncover common underlying associations across different conditions. Thus, a key observation from this review is the inherent variability within this population across multiple levels, including ID severity, age, and the varied expressions of mental health problems. However, this variability also introduces complexity. For instance, whereas ASD has a strong genetic component influenced by environmental factors, it differs significantly from behavioral problems, which are more directly associated with environmental factors [80, 81]. This contrast in underlying mechanisms may help explain our subgroup findings: externalizing behaviors were more often associated with SDOMH than ASD in the social/cultural and economic domains. In contrast, ASD showed relatively more associations in the demographic domain, possibly reflecting a different pattern of relationships. These findings suggest that social and economic environments are more consistently or directly associated with externalizing behavior problems, while the pathways linking SDOMH to ASD symptoms may be more complex or indirect. Other types of mental health problems could not be examined in this way due to the limited number of studies, highlighting an important direction for future research. Subgroup analyses by ID severity and age did not reveal any clear domain-specific patterns.

Despite the challenges posed by variability, some key patterns emerged. Overall, the review found that social, cultural, and economic factors were studied more frequently than demographic and neighborhood factors in research on mental health issues in youth with ID. Most evidence, both longitudinal and cross-sectional, was found in the social/cultural domain, particularly regarding associations between parental well-being, parenting behaviors, life events, and children's mental health. Specifically, studies found that in families where children exhibited more behavioral problems: (1) parents experienced higher distress or internalizing problems themselves, such as anxiety and depression; (2) parenting behaviors were less positive or more negative (i.e., harsh discipline was consistently associated with behavioral problems); and (3) children were exposed to stressful life events. These results align with a previous review [17], which already identified family dysfunction, parental stress and psychopathology as significant factors. This study reinforces and extends these insights by providing further empirical support within the context of children with ID. Viewed through the lens of the dual burden concept, these findings provide some support for one aspect of the concept: children with ID who experience more adverse SDOMH, particularly in the social/cultural domain, also exhibit higher levels of mental health problems. However, empirical comparative studies are needed to determine whether these adversities are more prevalent among children with ID and more strongly associated with mental health problems than in their typically developing peers.

Findings for SES and income within the economic domain of SDOMH have been inconsistent, aligning with previous reviews [16, 17]. This inconsistency is particularly evident in cross-sectional studies. Longitudinal studies have shown somewhat more consistent associations over time, but the limited number of longitudinal studies restricts conclusions about long-term effects. The variability in findings may stem from differences in SES measurement—such as income alone versus composite indicators— and that SES rarely operates in isolation. Low SES is often linked to poorer child mental health, particularly when combined with risks such as family adversity or parental psychological distress [82]. For example, education and income are closely related, and without multivariate analyses, their independent or combined relationship with mental health remains unclear. As a result, observed associations may be oversimplified, requiring cautious interpretation as they may not fully capture the multidimensional nature of SDOMH. Contextual factors, such as regional social support systems and income equality, may further influence SES-related findings across settings.

Although research in this area has expanded compared to previous reviews [16, 17], our findings reveal four significant gaps. First, while much research has focused on proximal family characteristics, broader social and environmental contexts—such as housing, neighborhood conditions, and community diversity—have received considerably less attention. This is underscored by the presence of only one study in the neighborhood domain, despite growing evidence linking neighborhood deprivation to poor mental health outcomes [79]. In addition, factors such as geographical remoteness, air pollution, and soil contamination remain underexamined, even though children with ID often live in disadvantaged areas where such risks are prevalent. Investigating these environmental factors is essential for a more comprehensive understanding of their mental health. A second key limitation in the current literature is the predominance of cross-sectional (86.1%) and univariate (75.5%) studies, which restricts causal inference and hinders the identification of the direction and relative importance of SDOMH over time. By focusing on isolated SDOMH, these studies often fail to account for confounding factors or explore potential mediating and moderating effects, thereby overlooking the complex interactions that are likely associated with mental health and limiting the reliability of findings within each domain. The narrow scope of most reviewed studies may partly account for inconsistent findings. To address these gaps, future research should prioritize longitudinal, multivariate designs that can more effectively capture the dynamics and interconnected nature of SDOMH. Third, while research has largely centred on identifying risk factors, protective factors have received considerably less attention. This highlights the need for a more balanced and comprehensive approach to research in this area. Fourth and final, this review found no qualitative studies, leaving significant gaps in understanding the subjective experiences and contextual nuances of SDOMH. Future research should incorporate qualitative methods to capture these experiences more comprehensively.

### Strengths and limitations

This review has several strengths. First, we minimized reporting bias by registering our protocol prospectively in PROSPERO. Second, we enhanced generalizability by including a wide range of mental health problems and ID severities. Third, we reduced selection bias through independent article screening by two researchers. Fourth, to enhance the reliability of the findings, two independent researchers critically appraised the individual studies and assessed the strength of evidence within each domain. Finally, the review offers a comprehensive overview of SDOMH across various domains, emphasizing environmental contexts and using an adapted version of Lund et al.'s (2018) framework [6]. This approach offers a holistic view of SDOMH’s role in youth with ID, deepening our understanding of the complex nature of these mental health problems and highlighting the need to consider broader social contexts, such as neighborhood, alongside individual factors.

Nonetheless, some limitations must be acknowledged as well. First, due to the exploratory nature of this review, as well as the diverse topics and varied findings of the included studies, a meta-analysis was not feasible. Second, focusing on Western populations combined with the inclusion of studies published only in English and Dutch, may have introduced selection bias, limiting the generalizability of findings to non-Western or developing countries. Lastly, it was beyond the scope of this review to examine the role of environmental events- such as conflict, displacement, and natural disasters— which may be key SDOMH in non-industrialized contexts [6].

### Meaning of this review

Despite the diversity and variability within this field of research, some patterns have emerged, particularly within the social/cultural domain. Within this domain, parental well-being, parenting behaviors, and exposure to stressful life events are key factors consistently linked to mental health problems in children with ID. These findings highlight the need for support systems addressing parental mental health and family stressors together with children’s mental health, moving beyond symptom-focused interventions. Therefore, we recommend a multidisciplinary, integrated family approach that strengthens collaboration between adult and child mental health services, providing comprehensive, intergenerational support tailored to the needs of families [83]. Equally important, however, are critical gaps in research—both with regard to the focus of and quality of studies. To advance this field, future research should adopt a more systematic approach, focusing on structured analyses of different SDOMH and their associations with varied mental health problems, using consistent measures. Moreover, longitudinal studies with mediating and moderating variables are essential to better understand the complex interplay between children’s mental health and their environment, to strengthen theoretical and empirical insights, and to improve the identification of specific at-risk groups.

## Supplementary Information

Below is the link to the electronic supplementary material.

Supplementary file1 (DOCX 15 KB)

Supplementary file2 (DOCX 22 KB)

Supplementary file3 (DOCX 40.1 KB)

Supplementary file4 (DOCX 19 KB)

Supplementary file5 (DOCX 19.3 KB)

Supplementary file6 (DOCX 25.2 KB)

## Data Availability

No datasets were generated or analysed during the current study.
