# Supplementary material for: Social determinants associated with mental health problems in youth with intellectual disability: a systematic literature review
Source: Eur Child Adolesc Psychiatry. 2025 Jul 1;34(12):3697–711. doi: 10.1007/s00787-025-02794-7 (PMC12743075; doi:10.1007/s00787-025-02794-7)
Supplement: Supplementary file 1 — Supplementary file1 (DOCX 15 KB) [file 787_2025_2794_MOESM1_ESM.docx]

**Appendix A. Categorization of SDOMH**

| *Domain* | Factor within SDOMH |
| --- | --- |
| Demographic | Parental age  Parental ethnicity  Population density |
| Economic | Household poverty  Health insurance coverage  Economic recessions  Survival  Economic inequality  Macroeconomic policy |
| Social/Cultural | Education  Literacy  Social support or isolation  Participation / activities  Life events  Parental education and literacy  Parental employment  Quality of family relationships  Well-being and health of family members  Social support, isolation or participation experienced by caregivers  Acceptance of the child  Social stability |
| Neighborhood | Exposure to crime  Access to playgrounds  Involvement in community events  Accessibility to public transportation Infrastructure  Neighborhood deprivation  Violence  Built environment |

*Note.* In accordance with the framework of Lund et al. (2018), (parental) education and employment are classified under the social/cultural domain rather than the economic domain.
