# Supplementary material for: Social determinants associated with mental health problems in youth with intellectual disability: a systematic literature review
Source: Eur Child Adolesc Psychiatry. 2025 Jul 1;34(12):3697–711. doi: 10.1007/s00787-025-02794-7 (PMC12743075; doi:10.1007/s00787-025-02794-7)
Supplement: Supplementary file 2 — Supplementary file2 (DOCX 22 KB) [file 787_2025_2794_MOESM2_ESM.docx]

**Appendix B. Search strategy**

**PubMed**

*Pubmed search strategy on September 5th, 2024: 3,733*

("Intellectual Disability"[Majr] OR "Intellectual disabilit*"[ti] OR "Mental Retardation"[ti] OR "Mentally Retarded"[ti] OR "Learning Disabilit*"[ti] OR "Intellectual Development Disorder*"[ti] OR "Mental Deficienc*"[ti] OR "Mentally Deficien*"[ti] OR "Developmental disabilit* "[ti] OR "Intellectual deficit*"[ti] OR "Persons with Mental Disabilities"[Majr] OR "mild mental"[tiab] OR "mild intellectual"[tiab] OR "borderline intellectual"[tiab])

AND ("Adolescent"[MeSH] OR "Child"[MeSH] OR "Young Adult"[MeSH] OR "Infant"[MeSH] OR "child*"[tw] OR "schoolchild*"[tw] OR "baby"[tw] OR "babies"[tw] OR "newborn*"[tw] OR "new-born*"[tw] OR "neonat*"[tw] OR "infant*"[tw] OR "infancy"[tw] OR "adolescen*"[tw] OR "boy"[tw] OR "boys"[tw] OR "boyhood"[tw] OR "girl"[tw] OR "girls"[tw] OR "girlhood"[tw] OR "youth"[tw] OR "youths"[tw] OR "toddler*"[tw] OR "teen"[tw] OR "teens"[tw] OR "teenage*"[tw] OR "Puberty"[Mesh] OR "puberty"[tw] OR "preschool"[tw] OR "pre school"[tiab] OR "pre-school"[tw] OR "juvenile"[tw] OR "juvenescence" [tw] OR "young"[tw] OR "youngster*"[tw] OR "kid"[tw] OR "kids"[tw] OR "underage*"[tw] OR "under age*"[tw] OR "puberal"[tw] OR "pubescent"[tw] OR "prepubescent"[tw] OR "prepuberty"[tw] OR "school age*"[tw] OR "schoolage*"[tw] OR "Pediatrics"[Mesh] OR "Pediatric*"[tw] OR "Paediatric*"[tw] OR "Pediatr*"[Journal] OR "Paediatr*"[Journal] OR ”undergraduate”[tw] OR “undergrad”[tw] OR “highschool”[tw] OR “high school”[tw] OR “secondary school”[tw] OR “college”[ti] OR “first-grader*” [tw] OR “second-grader*”[tw] OR “third-grader*”[tw] OR “fourth-grader*”[tw] OR “fifth-grader*”[tw] OR “sixth-grader*”[tw] OR “seventh-grader*”[tw] OR “freshman”[tw] OR “freshmen”[tw] OR “sophomore*”[tw])

AND ("Mental health problem*"[tiab] OR "Psychopatholog*"[tiab] OR "Mental health symptom*"[tiab] OR "Mental disorder*"[tiab] OR "Mental health disorder*"[tiab] OR "Psychiatric disorder*"[tiab] OR "Psychiatric diagnos*"[tiab] OR "Psychiatric morbid*"[tiab] OR "Behavior problem*"[tiab] OR "Behaviour problem*"[tiab] OR "Behavioral problem*"[tiab] OR "Behavioural problem*"[tiab] OR "Emotional problem*"[tiab] OR "Psychopathology"[majr] OR "Mental Disorders"[majr] OR “mental illness*”[tiab] OR “mentally ill”[tiab] OR “mental disease*”[tiab] OR “psychiatric disease*”[tiab] OR “psychiatric illness*”[tiab] OR “psychiatric symptom*”[tiab] OR “psychiatric sign*”[tiab] OR “behavior disorder*”[tiab] OR “problem behavio*”[tiab] OR “Child psychiatry”[mesh] OR “Adolescent psychiatry”[mesh] OR ”Behavioral Symptoms”[tiab] OR ”Impulsive Behavior”[mesh] OR ”Self-Injurious Behavior”[tiab] OR ”Obsessive Behavior”[tiab] OR ”Paranoid Behavior”[tiab] OR ”Problem Behavior”[tiab] OR "neuropsychiatric disease*"[tiab] OR "neuropsychiatric disorder*"[tiab] OR "neurodevelopmental disorder*"[tiab] OR "psychological disorder*"[tiab] OR "psychological disease*"[tiab] OR "psychological illness*"[tiab] OR "psychological disturbance"[tiab] OR "addiction*"[tiab] OR "adjustment disorder*"[tiab] OR "alexithymi*"[tiab] OR "anxiety disorder*"[tiab] OR "autism*"[tiab] OR "dissociative disorder"[tiab] OR "emotional disorder"[tiab] OR "Emotional Development*"[tiab] OR "Eating Disorders"[tiab] OR "mental instability"[tiab] OR "Mood Disorders"[tiab] OR "Obsessive-Compulsive Disorder"[tiab] OR "pathological lying"[tiab] OR "Personality Disorder*"[tiab] OR "psychosexual disorder"[tiab] OR "psychosis"[tiab] OR "psychotrauma"[tiab] OR "thought disorder"[tiab])

AND ("Social Factors"[majr] OR "Social Determinants of Health"[majr] OR "Home Environment"[Mesh] OR "Built Environment"[Mesh] OR "Vulnerable Populations"[Mesh] OR "Social"[ti] OR "divorce*"[ti] OR "Literacy"[ti] OR ”illiteracy”[ti] OR “illiterateness”[ti] OR "Poverty"[ti] OR “poverty”[Title] OR “poverty”[ot] OR "Unemploy* "[ti] OR "Deprivation "[ti] OR ”deprived”[ot] OR ”destitute”[ot] OR "maltreatment"[ti] OR "peer pressure"[ti] OR "Insurance"[ti] OR "Bully*"[ti] OR "Participation in activities"[ti] OR "engaging in activities"[ti] OR "inequalit*"[ti] OR "Health disparit*"[ti] OR "Health inequit* "[ti] OR "Risk* "[ti] OR "Cultural "[ti] OR "Protective "[ti] OR "Prevention"[ti] OR "Demographic"[ti] OR "Community diversity"[ti] OR "Population density"[ti] OR "Longevity"[ti] OR "Economic*"[ti] OR "Macroeconomic*"[ti] OR "Neighborhood "[ti] OR "Neighbourhood "[ti] OR "Infrastructure"[ti] OR "Built environment"[ti] OR "Environmental event*"[ti] OR "Family"[ti] OR "Families"[ti] OR "Socio-economic"[ti] OR "Socioeconomic"[ti] OR "minorit*"[ti] OR "Life event*"[ti] OR "Marital Status"[ti] OR "Population Group*"[ti] OR "Ethnic group*"[ti] OR "Married"[ti] OR "Separated"[ti] OR "Single Parent"[Mesh] OR "Single-Parent Family"[Mesh] OR "Single-Parent"[ti] OR "One-parent"[ti] OR "Employment"[Mesh:NoExp] OR "Employment"[ti] OR "Income*"[ti] OR "income*"[ot] OR “debt*”[ot] OR “indebted”[ot] OR "Affluence"[ti] OR "Disadvantaged"[ti] OR "Living standard*"[ti] OR "Marginal*"[ti] OR "Standard of living"[ti] OR "Financial difficult*"[ti] OR "Financial problem*"[ti] OR “Financial security”[ot] OR “Financial insecurity”[ot] OR "Occupation*"[ti] OR "Jobless"[ti] OR "Inequit*"[ti] OR "Job insecurity"[ti] OR "Workless"[ti] OR "Residence"[ti] OR "Safety"[ti] OR "Recreational facilit*"[ti] OR "Access to health service*"[ti] OR "Crime rate*"[ti] OR "Housing"[ti] OR "Psychosocial"[ti] OR "Psycho-social"[ti] OR "Education*"[ti] OR "Vulnerable Population*"[ti] OR "oppressed"[ti] OR "racial"[ti] OR "inequalit*"[ti] OR "injustice"[ti] OR "unrepresented"[ti] OR “underprivileged”[ot] OR “impoverish*”[Title] OR “impoverish*”[ot])

NOT ("mutation"[ot] OR "gene"[ot] OR "genes"[ot] OR "genetic"[ot] OR "mutation"[ti] OR "gene"[ti] OR "genes"[ti] OR "genetic"[ti])

**PsycINFO**

*PsycINFO search strategy on September 5th, 2024: 580*

(MM ("Intellectual Development Disorder" OR "Down's Syndrome") OR TI ("Intellectual disabilit*" OR "mental disabilit*" OR "Mental Retardation" OR "Mentally Retarded" OR "Learning Disabilit*" OR "Intellectual Development Disorder*" OR "Mental Deficien*" OR "Mentally Deficien*" OR "Developmental disabilit*" OR "Intellectual deficit*" OR "Persons with Mental Disabilities" OR "mild mental" OR "mild intellectual" OR "borderline intellectual")) AND (DE ("Puberty" OR "Pediatrics") OR TX ("child*" OR "schoolchild*" OR "baby" OR "babies" OR "newborn*" OR "new-born*" OR "neonat*" OR "infant*" OR "infancy" OR "adolescen*" OR "boy" OR "boys" OR "boyhood" OR "girl" OR "girls" OR "girlhood" OR "youth" OR "youths" OR "toddler*" OR "teen" OR "teens" OR "teenage*" OR "puberty" OR "preschool" OR "pre school" OR "pre-school" OR "juvenile" OR "young" OR "youngster*" OR "kid" OR "kids" OR "underage*" OR "under age*" OR "puberal" OR "pubescent" OR "prepubescent" OR "prepuberty" OR "school age*" OR "schoolage*" OR "Pediatric*" OR "Paediatric*" OR "juvenescence" OR "Pediatr*" OR "Paediatr*" OR ”undergraduate” OR “undergrad” OR “highschool” OR “high school” OR “secondary school” OR “college” OR “first-grader*” OR “second-grader*” OR “third-grader*” OR “fourth-grader*” OR “fifth-grader*” OR “sixth-grader*” OR “seventh-grader*” OR “freshman” OR “freshmen” OR “sophomore*”)) AND (TI ("Mental health problem*" OR "Psychopatholog*" OR "Mental health symptom*" OR "Mental disorder*" OR "Mental health disorder*" OR "Psychiatric disorder*" OR "Psychiatric diagnos*" OR "Psychiatric morbid*" OR "Behavior problem*" OR "Behaviour problem*" OR "Behavioral problem*" OR "Behavioural problem*" OR "Emotional problem*" OR "Psychopatholog*" OR MM ("Psychopathology" OR "Adolescent Psychopathology" OR "Child Psychopathology" OR "Mental Disorders" OR "Affective Disorders" OR "Anxiety Disorders" OR "Autism Spectrum Disorders" OR "Bipolar Disorder" OR "Borderline States" OR "Chronic Mental Illness" OR "Dissociative Disorders" OR "Eating Disorders" OR "Gender Dysphoria" OR "Mental Disorders due to General Medical Conditions" OR "Neurocognitive Disorders" OR "Neurosis" OR "Paraphilias" OR "Personality Disorders" OR "Psychosis" OR "Serious Mental Illness" OR "Sleep Wake Disorders" OR "Somatoform Disorders" OR "Substance Related and Addictive Disorders" OR "Thought Disturbances" OR “mental illness*” OR “mentally ill” OR “mental disease*” OR “psychiatric disease*” OR “psychiatric illness*” OR “psychiatric symptom*” OR “psychiatric sign*” OR “behavior disorder*” OR “problem behavio*” OR “Child psychiatry” OR “Adolescent psychiatry” OR ”Behavioral Symptoms” OR ”Impulsive Behavior” OR ”Self-Injurious Behavior” OR ”Obsessive Behavior” OR ”Paranoid Behavior” OR ”Problem Behavior” OR "neuropsychiatric disease*" OR "neuropsychiatric disorder*" OR "neurodevelopmental disorder*" OR "psychological disorder*" OR "psychological disease*" OR "psychological illness*" OR "psychological disturbance" OR "addiction*" OR "adjustment disorder*" OR "alexithymi*" OR "anxiety disorder*" OR "autism*" OR "dissociative disorder" OR "emotional disorder" OR "Emotional Development*" OR "Eating Disorders" OR "mental instability" OR "Mood Disorders" OR "Obsessive-Compulsive Disorder" OR "pathological lying" OR "Personality Disorder*" OR "psychosexual disorder" OR "psychosis" OR "psychotrauma" OR "thought disorder")) AND (MM ("Socioeconomic Factors" OR "Economic Disadvantage" OR "Home Environment" OR "Built Environment" OR "Vulnerable Populations" "Economic Resources" OR "Employment Status" OR "Income Level" OR "Social Class" OR "Social Disadvantage" OR "Socioeconomic Status" OR "Single Parents" OR "Single Fathers" OR "Single Mothers") OR TI ("Social" OR "divorce*" OR "Literacy" ”illiteracy” OR “illiterateness” OR "Poverty" OR "Unemploy* " OR "Deprivation " OR ”deprived” OR ”destitute” OR "maltreatment" OR "peer pressure" OR "Insurance" OR "Bully*" OR "Participation in activities" OR "engaging in activities" OR "inequalit*" OR "Health disparit*" OR "Health inequit* " OR "Risk* " OR "Cultural " OR "Protective " OR "Prevention" OR "Demographic" OR "Community diversity" OR "Population density" OR "Longevity" OR "Economic*" OR "Macroeconomic*" OR "Neighborhood " OR "Neighbourhood " OR "Infrastructure" OR "Built environment" OR "Environmental event*" OR "Family" OR "Families" OR "Socio-economic" OR "Socioeconomic" OR "minorit*" OR "Life event*" OR "Marital Status" OR "Population Group*" OR "Ethnic group*" OR "Married" OR "Separated" OR "Single-Parent" OR "One-parent" OR "Employment" OR "Income*" OR “debt*” OR “indebted” OR "Affluence" OR "Disadvantaged" OR "Living standard*" OR "Marginal*" OR "Standard of living" OR "living standard*" OR "Financial difficult*" OR "Financial problem*" OR “Financial security” OR “Financial insecurity” OR "Occupation*" OR "Jobless" OR "Inequit*" OR "Job insecurity" OR "Workless" OR "Residence" OR "Safety" OR "Recreational facilit*" OR "Access to health service*" OR "Crime rate*" OR "Housing" OR "Psychosocial" OR "Psycho-social" OR "Education*" OR "Vulnerable Population*" OR "oppressed" OR "racial" OR "inequalit*" OR "injustice" OR "unrepresented" OR “impoverish*” OR “impoverish*”)) NOT TI ("mutation" OR "gene" OR "genes" OR "genetic" OR "mutation" OR "gene" OR "genes" OR "genetic")

**Web of Science**

*Web of Science search strategy on September 5th, 2024: 351*

TI=("Intellectual disabilit*" OR "mental disabilit*" OR "Mental Retardation" OR "Mentally Retarded" OR "Learning Disabilit*" OR "Intellectual Development Disorder*" OR "Mental Deficien*" OR "Mentally Deficien*" OR "Developmental disabilit*" OR "Intellectual deficit*" OR "Down's Syndrome" OR "Down Syndrome" OR "Downs Syndrome" OR "mild mental" OR "mild intellectual" OR "borderline intellectual") AND TS=("child*" OR "schoolchild*" OR "baby" OR "babies" OR "newborn*" OR "new-born*" OR "neonat*" OR "infant*" OR "infancy" OR "adolescen*" OR "boy" OR "boys" OR "boyhood" OR "girl" OR "girls" OR "girlhood" OR "youth" OR "youths" OR "toddler*" OR "teen" OR "teens" OR "teenage*" OR "puberty" OR "preschool" OR "pre school" OR "pre-school" OR "juvenile" OR "young" OR "youngster*" OR "kid" OR "kids" OR "underage*" OR "under age*" OR "puberal" OR "pubescent" OR "prepubescent" OR "prepuberty" OR "school age*" OR "schoolage*" OR "Pediatric*" OR "Paediatric*" OR "juvenescence" OR "Pediatr*" OR "Paediatr*" OR "undergraduate" OR "undergrad" OR "highschool" OR "high school" OR "secondary school" OR "college" OR "first-grader*" OR "second-grader*" OR "third-grader*" OR "fourth-grader*" OR "fifth-grader*" OR "sixth-grader*" OR "seventh-grader*" OR "freshman" OR "freshmen" OR "sophomore*") AND TI=("Mental health problem*" OR "Psychopatholog*" OR "Mental health symptom*" OR "Mental disorder*" OR "Mental health disorder*" OR "Psychiatric disorder*" OR "Psychiatric diagnos*" OR "Psychiatric morbid*" OR "Behavior problem*" OR "Behaviour problem*" OR "Behavioral problem*" OR "Behavioural problem*" OR "Emotional problem*" OR "Psychopatholog*" OR "Affective Disorder*" OR "Anxiety Disorder*" OR "Autism Spectrum Disorder*" OR "Bipolar Disorder*" OR "Borderline State*" OR "Mental Illness" OR "Dissociative Disorder*" OR "Eating Disorder*" OR "Gender Dysphoria" OR "Neurocognitive Disorder*" OR "Neurosis" OR "Paraphilia*" OR "Personality Disorder*" OR "Psychosis" OR "Sleep Wake Disorder*" OR "Somatoform Disorder*" OR "Addict*" OR "Thought Disturbance*" OR ”mental illness*” OR “mentally ill” OR “mental disease*” OR “psychiatric disease*” OR “psychiatric illness*” OR “psychiatric symptom*” OR “psychiatric sign*” OR “behavior disorder*” OR “problem behavio*” OR “Child psychiatry” OR “Adolescent psychiatry” OR ”Behavioral Symptoms” OR ”Impulsive Behavior” OR ”Self-Injurious Behavior” OR ”Obsessive Behavior” OR ”Paranoid Behavior” OR ”Problem Behavior” OR "neuropsychiatric disease*" OR "neuropsychiatric disorder*" OR "neurodevelopmental disorder*" OR "psychological disorder*" OR "psychological disease*" OR "psychological illness*" OR "psychological disturbance" OR "addiction*" OR "adjustment disorder*" OR "alexithymi*" OR "anxiety disorder*" OR "autism*" OR "dissociative disorder" OR "emotional disorder" OR "Emotional Development*" OR "Eating Disorders" OR "mental instability" OR "Mood Disorders" OR "Obsessive-Compulsive Disorder" OR "pathological lying" OR "Personality Disorder*" OR "psychosexual disorder" OR "psychosis" OR "psychotrauma" OR "thought disorder") AND TI=("Income" OR "Social" OR "divorce*" OR "Literacy" OR "Poverty" OR "Unemploy* " OR "Deprivation " OR "maltreatment" OR "peer pressure" OR "Insurance" OR "Bully*" OR "Participation in activities" OR "engaging in activities" OR "inequalit*" OR "Health disparit*" OR "Health inequit*" OR "Risk*" OR "Cultural " OR "Protective" OR "Prevention" OR "Demographic" OR "Community diversity" OR "Population density" OR "Longevity" OR "Economic*" OR "Macroeconomic*" OR "Neighborhood " OR "Neighbourhood " OR "Infrastructure" OR "Built environment" OR "Environmental event*" OR "Family" OR "Families" OR "Socio-economic" OR "Socioeconomic" OR "minorit*" OR "Life event*" OR "Marital Status" OR "Population Group*" OR "Ethnic group*" OR "Married" OR "Separated" OR "Single-Parent*" OR "Single Parent*" OR "One-parent" OR "Employment" OR "Income*" OR "Affluence" OR "Disadvantaged" OR "Living standard*" OR "Marginal*" OR "Standard of living" OR "living standard*" OR "Financial difficult*" OR "Financial problem*" OR "Occupation*" OR "Jobless" OR "Inequit*" OR "Job insecurity" OR "Residence" OR "Safety" OR "Recreational facilit*" OR "Access to health service*" OR "Crime rate*" OR "Housing" OR "Psychosocial" OR "Psycho-social" OR "Education*" OR "Vulnerable Population*" OR "Literacy" OR "oppressed" OR "racial" OR "inequalit*" OR "injustice" OR "unrepresented" OR "Home Environment" OR "Built Environment" OR "Vulnerable Populations" OR ”illiteracy” OR “illiterateness” OR ”deprived” OR ”destitute” OR “debt*” OR “indebted” OR “Financial security” OR “Financial insecurity” OR “impoverish*” OR “impoverish*”) NOT TI=("mutation" OR "gene" OR "genes" OR "genetic" OR "mutation" OR "gene" OR "genes" OR "genetic")

**Cochrane**

*Cochrane search strategy on September 5th, 2024: 0*

("Intellectual disabilit*" OR "mental disabilit*" OR "Mental Retardation" OR "Mentally Retarded" OR "Learning Disabilit*" OR "Intellectual Development Disorder*" OR "Mental Deficien*" OR "Mentally Deficien*" OR "Developmental disabilit*" OR "Intellectual deficit*" OR "Down's Syndrome" OR "Down Syndrome" OR "Downs Syndrome"):ti AND ("child*" OR "schoolchild*" OR "baby" OR "babies" OR "newborn*" OR "new-born*" OR "neonat*" OR "infant*" OR "infancy" OR "adolescen*" OR "boy" OR "boys" OR "boyhood" OR "girl" OR "girls" OR "girlhood" OR "youth" OR "youths" OR "toddler*" OR "teen" OR "teens" OR "teenage*" OR "puberty" OR "preschool" OR "pre school" OR "pre-school" OR "juvenile" OR "young" OR "youngster*" OR "kid" OR "kids" OR "underage*" OR "under age*" OR "puberal" OR "pubescent" OR "prepubescent" OR "prepuberty" OR "school age*" OR "schoolage*" OR "Pediatric*" OR "Paediatric*"):ti,ab,kw AND ("Mental health problem*" OR "Psychopatholog*" OR "Mental health symptom*" OR "Mental disorder*" OR "Mental health disorder*" OR "Psychiatric disorder*" OR "Psychiatric diagnos*" OR "Psychiatric morbid*" OR "Behavior problem*" OR "Behaviour problem*" OR "Behavioral problem*" OR "Behavioural problem*" OR "Emotional problem*" OR "Psychopatholog*" OR "Affective Disorder*" OR "Anxiety Disorder*" OR "Autism Spectrum Disorder*" OR "Bipolar Disorder*" OR "Borderline State*" OR "Mental Illness" OR "Dissociative Disorder*" OR "Eating Disorder*" OR "Gender Dysphoria" OR "Neurocognitive Disorder*" OR "Neurosis" OR "Paraphilia*" OR "Personality Disorder*" OR "Psychosis" OR "Sleep Wake Disorder*" OR "Somatoform Disorder*" OR "Addict*" OR "Thought Disturbance*"):ti AND ("Income" OR "Social" OR "divorce*" OR "Literacy" OR "Poverty" OR "Unemploy* " OR "Deprivation " OR "maltreatment" OR "peer pressure" OR "Insurance" OR "Bully*" OR "Participation in activities" OR "engaging in activities" OR "inequalit*" OR "Health disparit*" OR "Health inequit*" OR "Risk*" OR "Cultural " OR "Protective" OR "Prevention" OR "Demographic" OR "Community diversity" OR "Population density" OR "Longevity" OR "Economic*" OR "Macroeconomic*" OR "Neighborhood " OR "Neighbourhood " OR "Infrastructure" OR "Built environment" OR "Environmental event*" OR "Family" OR "Families" OR "Socio-economic" OR "Socioeconomic" OR "minorit*" OR "Life event*" OR "Marital Status" OR "Population Group*" OR "Ethnic group*" OR "Married" OR "Separated" OR "Single-Parent*" OR "Single Parent*" OR "One-parent" OR "Employment" OR "Income*" OR "Affluence" OR "Disadvantaged" OR "Living standard*" OR "Marginal*" OR "Standard of living" OR "living standard*" OR "Financial difficult*" OR "Financial problem*" OR "Occupation*" OR "Jobless" OR "Inequit*" OR "Job insecurity" OR "Residence" OR "Safety" OR "Recreational facilit*" OR "Access to health service*" OR "Crime rate*" OR "Housing" OR "Psychosocial" OR "Psycho-social" OR "Education*" OR "Vulnerable Population*" OR "Literacy" OR "oppressed" OR "racial" OR "inequalit*" OR "injustice" OR "unrepresented"):ti NOT ("mutation" OR "gene" OR "genes" OR "genetic"):ti

**MEDLINE**

*MEDLINE search strategy on September 5th, 2024: 237*

(MM ("Intellectual Development Disorder" OR "Down's Syndrome") OR TI ("Intellectual disabilit*" OR "mental disabilit*" OR "Mental Retardation" OR "Mentally Retarded" OR "Learning Disabilit*" OR "Intellectual Development Disorder*" OR "Mental Deficien*" OR "Mentally Deficien*" OR "Developmental disabilit*" OR "Intellectual deficit*" OR "Persons with Mental Disabilities" OR "mild mental" OR "mild intellectual" OR "borderline intellectual")) AND (DE ("Puberty" OR "Pediatrics") OR TX ("child*" OR "schoolchild*" OR "baby" OR "babies" OR "newborn*" OR "new-born*" OR "neonat*" OR "infant*" OR "infancy" OR "adolescen*" OR "boy" OR "boys" OR "boyhood" OR "girl" OR "girls" OR "girlhood" OR "youth" OR "youths" OR "toddler*" OR "teen" OR "teens" OR "teenage*" OR "puberty" OR "preschool" OR "pre school" OR "pre-school" OR "juvenile" OR "young" OR "youngster*" OR "kid" OR "kids" OR "underage*" OR "under age*" OR "puberal" OR "pubescent" OR "prepubescent" OR "prepuberty" OR "school age*" OR "schoolage*" OR "Pediatric*" OR "Paediatric*" OR "juvenescence" OR "Pediatr*" OR "Paediatr*" OR ”undergraduate” OR “undergrad” OR “highschool” OR “high school” OR “secondary school” OR “college” OR “first-grader*” OR “second-grader*” OR “third-grader*” OR “fourth-grader*” OR “fifth-grader*” OR “sixth-grader*” OR “seventh-grader*” OR “freshman” OR “freshmen” OR “sophomore*”)) AND (TI ("Mental health problem*" OR "Psychopatholog*" OR "Mental health symptom*" OR "Mental disorder*" OR "Mental health disorder*" OR "Psychiatric disorder*" OR "Psychiatric diagnos*" OR "Psychiatric morbid*" OR "Behavior problem*" OR "Behaviour problem*" OR "Behavioral problem*" OR "Behavioural problem*" OR "Emotional problem*" OR "Psychopatholog*" OR MM ("Psychopathology" OR "Adolescent Psychopathology" OR "Child Psychopathology" OR "Mental Disorders" OR "Affective Disorders" OR "Anxiety Disorders" OR "Autism Spectrum Disorders" OR "Bipolar Disorder" OR "Borderline States" OR "Chronic Mental Illness" OR "Dissociative Disorders" OR "Eating Disorders" OR "Gender Dysphoria" OR "Mental Disorders due to General Medical Conditions" OR "Neurocognitive Disorders" OR "Neurosis" OR "Paraphilias" OR "Personality Disorders" OR "Psychosis" OR "Serious Mental Illness" OR "Sleep Wake Disorders" OR "Somatoform Disorders" OR "Substance Related and Addictive Disorders" OR "Thought Disturbances" OR “mental illness*” OR “mentally ill” OR “mental disease*” OR “psychiatric disease*” OR “psychiatric illness*” OR “psychiatric symptom*” OR “psychiatric sign*” OR “behavior disorder*” OR “problem behavio*” OR “Child psychiatry” OR “Adolescent psychiatry” OR ”Behavioral Symptoms” OR ”Impulsive Behavior” OR ”Self-Injurious Behavior” OR ”Obsessive Behavior” OR ”Paranoid Behavior” OR ”Problem Behavior” OR "neuropsychiatric disease*" OR "neuropsychiatric disorder*" OR "neurodevelopmental disorder*" OR "psychological disorder*" OR "psychological disease*" OR "psychological illness*" OR "psychological disturbance" OR "addiction*" OR "adjustment disorder*" OR "alexithymi*" OR "anxiety disorder*" OR "autism*" OR "dissociative disorder" OR "emotional disorder" OR "Emotional Development*" OR "Eating Disorders" OR "mental instability" OR "Mood Disorders" OR "Obsessive-Compulsive Disorder" OR "pathological lying" OR "Personality Disorder*" OR "psychosexual disorder" OR "psychosis" OR "psychotrauma" OR "thought disorder")) AND (MM ("Socioeconomic Factors" OR "Economic Disadvantage" OR "Home Environment" OR "Built Environment" OR "Vulnerable Populations" "Economic Resources" OR "Employment Status" OR "Income Level" OR "Social Class" OR "Social Disadvantage" OR "Socioeconomic Status" OR "Single Parents" OR "Single Fathers" OR "Single Mothers") OR TI ("Social" OR "divorce*" OR "Literacy" ”illiteracy” OR “illiterateness” OR "Poverty" OR "Unemploy* " OR "Deprivation " ”deprived” OR ”destitute” OR "maltreatment" OR "peer pressure" OR "Insurance" OR "Bully*" OR "Participation in activities" OR "engaging in activities" OR "inequalit*" OR "Health disparit*" OR "Health inequit* " OR "Risk* " OR "Cultural " OR "Protective " OR "Prevention" OR "Demographic" OR "Community diversity" OR "Population density" OR "Longevity" OR "Economic*" OR "Macroeconomic*" OR "Neighborhood " OR "Neighbourhood " OR "Infrastructure" OR "Built environment" OR "Environmental event*" OR "Family" OR "Families" OR "Socio-economic" OR "Socioeconomic" OR "minorit*" OR "Life event*" OR "Marital Status" OR "Population Group*" OR "Ethnic group*" OR "Married" OR "Separated" OR "Single-Parent" OR "One-parent" OR "Employment" OR "Income*" OR “debt*” OR “indebted” OR "Affluence" OR "Disadvantaged" OR "Living standard*" OR "Marginal*" OR "Standard of living" OR "living standard*" OR "Financial difficult*" OR "Financial problem*" “Financial security” OR “Financial insecurity” OR "Occupation*" OR "Jobless" OR "Inequit*" OR "Job insecurity" OR "Workless" OR "Residence" OR "Safety" OR "Recreational facilit*" OR "Access to health service*" OR "Crime rate*" OR "Housing" OR "Psychosocial" OR "Psycho-social" OR "Education*" OR "Vulnerable Population*" OR "oppressed" OR "racial" OR "inequalit*" OR "injustice" OR "unrepresented" OR “impoverish*” OR “impoverish*”)) NOT TI ("mutation" OR "gene" OR "genes" OR "genetic" OR "mutation" OR "gene" OR "genes" OR "genetic")
