# Supplementary material for: Social determinants associated with mental health problems in youth with intellectual disability: a systematic literature review
Source: Eur Child Adolesc Psychiatry. 2025 Jul 1;34(12):3697–711. doi: 10.1007/s00787-025-02794-7 (PMC12743075; doi:10.1007/s00787-025-02794-7)
Supplement: Supplementary file 3 — Supplementary file3 (DOCX 40.1 KB) [file 787_2025_2794_MOESM3_ESM.docx]

**Appendix C. Study characteristics**

| **Study number** | **Author (year)** | **Country** | **Design** | **Sample size number with ID** | **Age-based stages** | **Severity of ID** | **Type of mental health problems** | **SDOMH** | **Quality of the study** |
| --- | --- | --- | --- | --- | --- | --- | --- | --- | --- |
| [28] | Schieve, Clayton, Durkin, Wingate, and Drews-Botsch (2015) | USA | Cross-sectional | 4821 | Middle childhood | Severe + moderate + mild | ASD | Maternal race/ethnicity, Maternal education, Maternal age, Mother unmarried at birth | High |
| [29] | Dekker and Koot (2003) | The Netherlands | Longitudinal | 474 | Middle childhood + early adolescence + late adolescence | Severe + moderate + mild + borderline | Anxiety, Disruptive Disorder, Mood Disorder, Psychopathology | Psychopathology of primary caregiver, Parental referral to mental health care, Sibling referral to mental health care, Family dysfunction, Low parental educational level, Non-Dutch parents, Single parent, Low SES, Negative life events | High |
| [30] | Eisenhower and Blacher (2006) | USA | Cross-sectional | 226 | Late adolescence | Severe + moderate | Behavior Problems | Employment status, Marital status, Ethnic group | Low |
| [31] | Hatton & Emerson (2009) | UK | Cross-sectional | 123 | Early childhood + middle childhood + early adolescence + late adolescence | Severe | Behavior Problems | Ethnicity of the mother, Parental anxiety, Parental distress, Parental health, Parental depression | Low |
| [32] | Morinaga, Hollander, Heuvelman, Lundberg, Dalman, Rai, and Magnusson (2021) | Sweden | Longitudinal | 8857 | Early childhood + middle childhood + early adolescence | Unknown | ASD | Timing of Child's Birth in Relation to Maternal Migration | High |
| [33] | Akdemir, Pehlivantürk, Ünal, and Özusta (2009) | Turkey | Cross-sectional | 37 | Early childhood | Mild + borderline | ASD | Parents level of education, Parental employment statement, Maternal age, Paternal age | Low |
| [34] | Avci (2024) | Turkey | Cross-sectional | 91 | Early adolescence + late adolescence | Mild | Emotional Problems, Conduct Problems, Hyperactivity | Birth order, Maternal age, Mothers education level, Fathers education level, Perceived social support, | High |
| [35] | Emerson and Brigham (2015) | UK | Cross-sectional | 2236 | Unknown | Unknown | Behavior Problems | Socio-economic position, Parent in care or abused, One parent family, Parent under 18, 3+ children under five, Separation/divorce, Family bereavement, Violence within family, Parent abuses alcohol, Parent abuses drugs, Parenting difficulties, Parental mental health | Medium |
| [36] | Pinborough-Zimmerman, Bilder, Bakian, Satterfield, Carbone, Nangle, Randall, and McMahon (2011) | USA | Longitudinal | 245 | Middle childhood | Unknown | ASD | Income, Federal taxes paid, Tax exemptions | Medium |
| [37] | Baker, Neece, Fenning, Crnic, and Blacher (2010) | USA | Longitudinal | 236 | Early childhood | Moderate + mild + borderline | ADHD | Maternal Education, Family Income (% > 50 K), Scaffolding, Positive Parenting, Negative Parenting, Dyadic pleasure, Dyadic conflict | High |
| [38] | Baker and Blacher (2021) | USA | Cross-sectional (part of longitudinal study) | 161 | Early adolescence | Moderate + mild + borderline | ASD | Maternal education, Maternal employment, Maternal marital status, Family income, Maternal anxiety, Maternal depression, Maternal hostility, Maternal Interpersonal Sensitivity, Maternal Somatization | Low |
| [39] | Baker, Seltzer, and Greenberg (2012) | USA | Cross-sectional | 115 | Early adolescence + late adolescence | Unknown | ASD, Behavior Problems | Maternal internalising symptoms, Marital satisfaction, Family cohesion, Income, Maternal education | Medium |
| [40] | Chadwick, Kusel, and Cuddy (2008) | UK | Longitudinal | 82 | Early adolescence | Severe | Externalising Problems, Overactivity, Destructive Behavior, Self-Injury | Family’s housing tenure, Single parent, No maternal educational qualifications, Main source of household income, Lack of continuity in maternal care, Lack of continuity in paternal care, Expressed parental criticism, Expressed parental warmth, Disciplinary aggression | High |
| [41] | Emerson and Hatton (2007) | UK | Cross-sectional | 10438 | Middle childhood + early adolescence | Unknown | Conduct Disorder, Emotional Disorder | Household income, Occupational prestige, Maternal education, Single parenthood, Household income, Occupational prestige, Maternal education, Single parenthood | High |
| [42] | Williams, Bailey, and Hastings (2022) | UK | Cross-sectional | 574 | Middle childhood + early adolescence | Unknown | Behavior Problems | Household poverty, Maternal life satisfaction, Maternal distress | High |
| [43] | Quine (1986) | UK | Cross-sectional | 200 | Early childhood + middle childhood | Severe | Behavior Problems | Single parent, Family size, Financial help | Low |
| [44] | Dworschak, Ratz, and Wagner (2016) | Germany | Cross-sectional | 1629 | Middle childhood + early adolescence + late adolescence | Severe + moderate + mild + borderline | Challenging Behavior | SES | Medium |
| [45] | Saunders, Tilford, Fussell, Schulz, Casey, and Kuo (2015) | USA | Cross-sectional | 1983 | Early childhood + middle childhood + early adolescence | Unknown | ASD | Health insurance coverage | Low |
| [46] | Schuiringa, van Nieuwenhuijzen, Orobio de Castro, and Matthys (2015) | The Netherlands | Cross-sectional | 113 | Middle childhood + early adolescence | Mild + borderline | Behavior Problems | SES, Involvement, Positive parenting scale, Monitoring, Positive discipline, Physical punishment, Rules, Acceptance, Sense of competence, Closeness | High |
| [47] | Baker, Blacher, and Pfeiffer (1993) | USA | Cross-sectional | 234 | Middle childhood + early adolescence + late adolescence | Severe + moderate + mild | Psychopathology | Parents marital status, Family socioeconomic status | Low |
| [48] | Kimura and Yamazaki (2016) | Japan | Cross-sectional | 613 | Early childhood + middle childhood + early adolescence + late adolescence | Unknown | ASD | Employment status, Marital status, Standard of living | Low |
| [49] | Scambler, Hepburn, Hagerman, and Rogers (2007) | USA | Cross-sectional | 17 | Early childhood | Unknown | ASD | SES | Medium |
| [50] | Weiss, Ting, and Perry (2016) | Canada | Cross-sectional | 141 | Middle childhood + early adolescence | Severe | Psychopathology, Maladaptive Behavior | Parental mental health problems, Family quality of life, Total negative life events, Financial Hardship | Low |
| [51] | Baker, McIntyre, Blacher, Crnic, Edelbrock, and Low (2003) | USA | Longitudinal | 82 | Early childhood | Moderate + mild | Behavior Problems | Parental stress level | Medium |
| [52] | Hall, Burns, and Reiss (2007) | USA | Cross-sectional | 150 families | Middle childhood | Moderate + mild | Behavior Problems | Maternal distress | Medium |
| [53] | Long, Kao, Plante, Seifer, and Lobato (2015) | USA | Cross-sectional | 192 | Middle childhood | Severe + moderate + mild + borderline | Maladaptive Behavior | Overall maternal distress, Maternal despressive symptoms, Maternal somatic symptoms | Medium |
| [54] | Neece and Baker (2008) | USA | Longitudinal, 2 years | 189 | Middle childhood | Moderate + mild + borderline | Behavior Problems | Maternal parenting stress | Medium |
| [55] | Robinson and Neece (2015) | USA | Cross-sectional | 44 | Early childhood | Moderate + mild | Behavior Problems | Marital satisfaction, Parental distress | Medium |
| [56] | Staunton, Kehoe, and Sharkey (2020) | Ireland | Cross-sectional | 33 | Middle childhood + early adolescence | Moderate | Challenging Behaviors, ASD | Parental psychological stress, Family quality of life, Level of support | Low |
| [57] | Bailey, Totsika, Hastings, Hatton, and Emerson (2019) | UK | Longitudinal cohort study | 555 | Early childhood + middle childhood | Unknown | Internalising and Externalising Problems | Maternal life satisfaction, Maternal distress | High |
| [58] | Hastings, Daley, Burns, and Beck (2006) | UK | Longitudinal | T0: 75; T1: 56 | Early childhood + middle childhood + early adolescence + late adolescence | Unknown | Internalising and Externalising Problems | Maternal depression, Maternal criticism, Maternal anxiety, Maternal distress, Maternal criticism | Low |
| [59] | Wallander, Dekker, and Koot (2006) | The Netherlands | Longitudinal | 474 | Middle childhood + early adolescence | Severe + moderate + mild + borderline | Psychopathology, Internalising and Externalising Problems | Parental distress, Parents mental health treatment history, Family dysfunction, Life events exposure, Low SES, Single parent | High |
| [60] | Embregts, du Bois, and Graef (2010) | The Netherlands | Cross-sectional | 45 | Early adolescence | Mild + borderline | Behavior Problems | Parents sense of competence, Restriction of role, Attachment, Parental depression, Parents health, Parental social isolation, Relationship with spouse, Negative life events, Positive life experiences | Low |
| [61] | Kobe and Hammer (1994) | USA | Cross-sectional | 29 | Early childhood + middle childhood | Severe + moderate + mild | Depression | Maternal depression, Attachment, Restriction of role, Sense of competence, Parental social isolation, Relationship with spouse, Parental health | Low |
| [62] | Stewart, Dave, and Lapshina (2023) | Canada | Cross-sectional | 517 | Middle childhood + early adolescence | Severe + moderate + mild + borderline | Psychiatric Diagnosis | Parental mental health difficulties, Sibling mental health difficulties, Traumatic life events | High |
| [63] | McCarthy (2008) | UK | Cross-sectional | 193 | Middle childhood + early adolescence | Unknown | Severe Behavior Disorder | Quality of marriage, Parental mental health, Social class | Low |
| [64] | Smith, Hong, Greenberg, and Mailick (2016) | USA | Longitudinal | 147 | Late adolescence | Unknown | Behavior Problems, Internalising, Externalising Problems, ASD | Maternal depression, Criticism, Warmth | Low |
| [65] | Lapshina and Stewart (2021) | Canada | Cross-sectional | 502 | Middle childhood + early adolescence | Severe + moderate + mild + borderline | Externalising Problems | Sexual assault/abuse, Physical assault/abuse, Emotional abuse, Witnessed domestic violence, Parental addiction, Death of a parent, Death in family, Parental abandonment, Violent neighborhood, Witnessed severe accident (disaster, terrorism, violence, or abuse) | High |
| [66] | Emerson (2003) | UK | Cross-sectional | 264 | Middle childhood + early adolescence | Unknown | Conduct Disorder, ADHD, Emotional Disorder, Anxiety Disorder, Depression, ASD, Psychopathology | Head of household classified in partly skilled and unskilled occupations, Household income, Single parents, Psychiatric morbidity among carers, Patterns of family functioning, Use of punitive strategies, Potentially stressful life events | Medium |
| [67] | Hatton, Emerson, Robertson, and Baines (2018) | UK | Longitudinal cohort study | 572 | Early adolescence + late adolescence | Moderate + mild | Psychopathology | Workless household, Single-parent household | High |
| [68] | Tonge and Einfeld (2003) | Australia | Longitudinal | 976 | Middle childhood + early adolescence | Severe + moderate + mild | Psychopathology | Family employment status, Social class, Living in nonfamily care | Low |
| [69] | Koskentausta, Iivanainen, and Almqvist (2007) | Finland | Cross-sectional | 75 | Middle childhood | Severe + moderate + mild | Psychopathology | SES, Number of children in the family, Birth order | Medium |
| [70] | Williams, Thompson, Hayden, and Hastings (2024) | UK | Longitudinal | 296 | Middle childhood + early adolescence | Moderate + mild | Behavior Problems | Birth order, Sibling warmth, Sibling conflict | High |
| [71] | Margalit, Shulman, and Stuchiner (1989) | Israel | Cross-sectional | 39 | Middle childhood + early adolescence | Moderate | Disruptive Behavioral Problems | Family Environmental Scale (Cohesion), Family Environmental Scale (Conflict) | Low |
| [72] | Boehm and Carter (2019) | USA | Cross-sectional | 529 | Early childhood + middle childhood + early adolescence + late adolescence | Severe + moderate + mild | ASD | Family relationships, Friend relationships | High |
| [73] | Totsika, Hastings, Emerson, and Hatton (2020) | UK | Longitudinal | 555 | Early childhood + middle childhood + early adolescence | Moderate + mild | Emotional Problems, Hyperactivity, Conduct Problems, Behavior Problems | Positive relationship, Adversarial parenting | High |
| [74] | McDonnell, Boan, Bradley, Seay, Charles, and Carpenter (2019) | USA | Cross-sectional | 4988 | Early childhood | Severe + moderate + mild | ASD | Overall maltreatment | High |
| [75] | Hemm, Dagnan, and Meyer (2018) | UK | Cross-sectional | 21 | Late adolescence | Mild + borderline | Anxiety | Parental overprotection | Medium |
| [76] | Van Rest, Van Nieuwenhuijzen, Kupersmidt, Vriens, Schuengel, and Matthys (2020) | The Netherlands | Cross-sectional | 220 | Early adolescence | Mild + borderline | Externalising Problems | SES | High |
| [77] | Ghaziuddin, Alessi, and Greden (1995) | USA | Cross-sectional | 22 | Middle childhood + early adolescence + late adolescence | Mild + borderline | Depression | Mean Life Events Score | Low |
| [78] | Foley, Jacoby, Einfeld, Girdler, Bourke, Riches, and Leonard (2014) | Australia | Longitudinal | T0: 118; T1: 103 | Late adolescence | Unknown | Behavior Problems | Day occupations: open employment, Day occupations: training, Day occupations: sheltered employment, Day occupations: day recreation programs | Medium |
