# Supplementary material for: Social determinants associated with mental health problems in youth with intellectual disability: a systematic literature review
Source: Eur Child Adolesc Psychiatry. 2025 Jul 1;34(12):3697–711. doi: 10.1007/s00787-025-02794-7 (PMC12743075; doi:10.1007/s00787-025-02794-7)
Supplement: Supplementary file 4 — Supplementary file4 (DOCX 19 KB) [file 787_2025_2794_MOESM4_ESM.docx]

**Appendix D. AXIS quality appraisal criteria and assigned weights**

*Note.* (R) = reversed. Items 13 and 19 are reverse-coded; a ‘no’ response on these items was considered a positive indicator of quality.

| **No.** | **Question** | **Max. weight** |
| --- | --- | --- |
| 1 | Were the aims/objectives of the study clear? | 1.5 |
| 2 | Was the study design appropriate for the stated aim(s)? | 2 |
| 3 | Was the sample size justified? | 1 |
| 4 | Was the target/reference population clearly defined? (Is it clear who the research was about?) | 1 |
| 5 | Was the sample frame taken from an appropriate population base so that it closely represented the target/reference population under investigation? | 1 |
| 6 | Was the selection process likely to select subjects/participants that were representative of the target/reference population under investigation? | 1 |
| 7 | Were measures undertaken to address and categorise non-responders? | 1 |
| 8 | Were the risk factor and outcome variables measured appropriate to the aims of the study? | 2 |
| 9 | Were the risk factor and outcome variables measured correctly using instruments/measurements that had been trialled, piloted or published previously? | 2 |
| 10 | Is it clear what was used to determined statistical significance and/or precision estimates? (e.g. p-values, confidence intervals) | 1 |
| 11 | Were the methods (including statistical methods) sufficiently described to enable them to be repeated? | 2 |
| 12 | Were the basic data adequately described? | 1 |
| 13 (R) | Does the response rate raise concerns about non-response bias? | 1 |
| 14 | If appropriate, was information about non-responders described? | 1 |
| 15 | Were the results internally consistent? | 1 |
| 16 | Were the results presented for all the analyses described in the methods? | 2 |
| 17 | Were the authors' discussions and conclusions justified by the results? | 1.5 |
| 18 | Were the limitations of the study discussed? | 1 |
| 19  (R) | Were there any funding sources or conflicts of interest that may affect the authors’ interpretation of the results? | 1 |
| 20 | Was ethical approval or consent of participants attained? | 1 |
