# Supplementary material for: Social determinants associated with mental health problems in youth with intellectual disability: a systematic literature review
Source: Eur Child Adolesc Psychiatry. 2025 Jul 1;34(12):3697–711. doi: 10.1007/s00787-025-02794-7 (PMC12743075; doi:10.1007/s00787-025-02794-7)
Supplement: Supplementary file 5 — Supplementary file5 (DOCX 19.3 KB) [file 787_2025_2794_MOESM5_ESM.docx]

**Appendix E. Extended findings**

| **Domain** | **Size of evidence (no. of studies and % of total studies)** | **Quality (individual studies)** | **Consistency of findings** | **Context** | **Perspective** | **Overall strength of evidence** |
| --- | --- | --- | --- | --- | --- | --- |
| Demographic | n = 8 (15.7%)  [28–35] | High: 4  Medium: 1  Low: 3 | Inconsistent | Specific (n = 3) Mixed (n = 5) | Single (n = 7) Multiple (n = 1) | Medium |
| Economic | n = 17 (33.3%)  [35–50, 66] | High: 5  Medium: 6  Low: 6 | Contradictory | Specific (n = 9) Mixed (n = 8) | Single (n = 13) Multiple (n = 4) | Medium |
| Social / cultural | n = 46 (90.2%)  [28–31, 33–35, 37–43, 46–48, 50–78] | High: 19  Medium: 11  Low: 17 | Contradictory | Specific (n = 18) Mixed (n = 29) | Single (n = 34) Multiple (n = 13) | Strong |
| Neighborhood | n = 1 (1.2%)  [65] | High: 1  Medium: 0  Low: 0 | N.A. | Specific (n = 0) Mixed (n=1) | Single (n = 1) Multiple (n = 0) | N.A. |

*Note.* N.A. = not applicable. Size of evidence = number of studies and percentage of total included studies; Quality = distribution of study quality ratings (high/medium/low); Consistency = extent to which findings align across studies (e.g., consistent, inconsistent, contradictory); Context = whether findings were observed in narrowly defined samples (e.g., specific diagnoses or subgroups) versus broader or more diverse populations (specific vs. mixed); Perspective = number of informants contributing to the evidence (single vs. multiple); Overall strength of evidence reflects a cumulative rating of the five criteria (see Methods for full definitions).
