# Supplementary material for: Social determinants associated with mental health problems in youth with intellectual disability: a systematic literature review
Source: Eur Child Adolesc Psychiatry. 2025 Jul 1;34(12):3697–711. doi: 10.1007/s00787-025-02794-7 (PMC12743075; doi:10.1007/s00787-025-02794-7)
Supplement: Supplementary file 6 — Supplementary file6 (DOCX 25.2 KB) [file 787_2025_2794_MOESM6_ESM.docx]

**Appendix F. Summary of results categorized by domain and subgroup**

**Table 3.** Severity of ID

| **Domain** | **Severe** | **Severe + Moderate** | **Moderate** | **Moderate + Mild + Borderline** | **Mild** | **Mild + Borderline** | **Entire spectrum** | **Unknown** |
| --- | --- | --- | --- | --- | --- | --- | --- | --- |
| *n* | *4* | *1* | *2* | *9* | *1* | *7* | *13* | *15* |
| *Demographic* | 0/1 | 0/1 | - | - | 1/1 | 0/1 | 1/2 | 1/2 |
| *Economic* | 1/3 | - | - | 0/2 |  | 0/1 | 1/2 | 4/9 |
| *Social/Cultural* | 4/4 | 0/1 | 1/2 | 7/9 | 1/1 | 5/6 | 10/12 | 10/11 |
| *Neighborhood* | - | - | - | - |  | - | 1/1 | - |

*Note.* The first number in each cell represents the number of studies that found a significant result, while the number after the slash indicates the total number of studies.

**Table 4.** Age groups

| **Domain** | **EC** | **﻿ EC + MC** | **MC** | **MC + EA** | **EA** | **EA + LA** | **LA** | **Combination** | **Unknown** |
| --- | --- | --- | --- | --- | --- | --- | --- | --- | --- |
| *n* | *6* | *3* | *6* | *13* | *4* | *3* | *4* | *11* | *1* |
| *Demographic* | 0/1 | - | 1/1 | - | - | 1/1 | 0/1 | 1/3 | 0/1 |
| *Economic* | 0/2 | 1/1 | 1/1 | 2/5 | 0/1 | 0/1 | - | 1/4 | 1/1 |
| *Social/Cultural* | 3/5 | 3/3 | 5/5 | 9/12 | 4/4 | 2/3 | 3/4 | 7/7 | 1/1 |
| *Neighborhood* | - | - | - | 1/1 | - | - | - | - | - |

*Note.* EC = Early childhood; MC = Middle childhood; EA = Early adolescence; LA = Late adolescence. The first number in each cell represents the number of studies that found a significant result, while the number after the slash indicates the total number of studies.

| **Domain** | **ASD** | **Externalizing problems^a^** |
| --- | --- | --- |
| *n* | *14* | *31* |
| *Study numbers:* | *28, 32, 33, 36, 38, 39*, 45, 48, 49, 56*, 64, 66*, 72, 74* | *29, 30, 31, 34, 35, 37, 39*, 42, 43, 44, 46, 50, 51, 52, 53, 54, 55, 56*, 57, 58, 59, 60, 63, 64, 65, 66, 70, 71, 73, 76, 78* |
| *Demographic* | *2/3* | *1/4* |
| *Economic* | *2/7* | *4/8* |
| *Social/Cultural* | *7/11* | *27/30* |
| *Neighborhood* | - | 1/1 |

**Table 5.** Mental health conditions (sub-analysis)

*Note.* The first number in each cell represents the number of studies that found a significant result, while the number after the slash indicates the total number of studies. *Study included in both mental health conditions. ^a^outward-directed behaviors such as behavioral problems, aggression, hyperactivity, or conduct issues.
